# Occurrence of *Echinococcus* *granulosus* *sensu* *lato* and Other Taeniids in Bhutan

**DOI:** 10.3390/pathogens10030330

**Published:** 2021-03-11

**Authors:** Puspa M. Sharma, Nirmal K. Thapa, Pema Tshomo, Tshewang Dema, Cristian A. Alvarez Rojas, Tenzin Tenzin, Ratna B. Gurung, Tshering Norbu, Lhatru Lhatru, Phurpa Namgyel, Chimi Jamtsho, Kinzang Dukpa, Yoenten Phuentshok, Krishna P. Sharma, Sonam Pelden, Peter Deplazes

**Affiliations:** 1 National Centre for Animal Health, Department of Livestock, Ministry of Agriculture and Forests, Serbithang, Thimphu 11001, Bhutan; nkthapa08@hotmail.com (N.K.T.); tenden2012@gmail.com (P.T.); karmajigme24@yahoo.com (T.D.); tenzinvp@gmail.com (T.T.); rgur1038@uni.sydney.edu.au (R.B.G.); kinduk2009@gmail.com (K.D.); 2 Institute of Parasitology, University of Zurich, 8057 Zurich, Switzerland; cristian.alvarezrojas@uzh.ch; 3 Livestock Extension Centre, Department of Livestock, Ministry of Agriculture and Forests, Sakteng, Trashigang 42005, Bhutan; tsheringtn2@gmail.com; 4 Livestock Extension Centre, Department of Livestock, Ministry of Agriculture and Forests, Merak, Trashigang 42005, Bhutan; lhatru@gmail.com; 5 Livestock Extension Centre, Department of Livestock, Ministry of Agriculture and Forests, Thangbi, Choekhor, Bumthang 32001, Bhutan; namgyelphurpa@gmail.com; 6 Department of Livestock, District Veterinary Hospital, Ministry of Agriculture and Forests, Trashiyangtse 46001, Bhutan; chimi.jamtsho@yahoo.com; 7 Phuentshok Epi Solutions, Lhakhangdoma, Phaktakha, Athang, Wangdue Phodrang 14001, Bhutan; vetyoen@gmail.com; 8 Jigme Dorji Wangchuck National Referral Hospital, Ministry of Health, Thimphu 11001, Bhutan; drukpath@gmail.com; 9 Royal Centre for Disease Control, Department of Public Health, Ministry of Health, Serbithang, Thimphu 11001, Bhutan; spelden@health.gov.bt

**Keywords:** cystic echinococcosis, *E. ortleppi*, coenurosis, *Taenia multiceps*

## Abstract

The present research shows the results of a national study documenting the occurrence and genetic diversity of *Echinococcus* and *Taenia* species across Bhutan. Environmental dog faecal samples (n = 953) were collected from 2016 to 2018 in all 20 Bhutanese districts, mainly in urbanised areas. Cystic echinococcosis cysts were isolated from 13 humans and one mithun (*Bos frontalis*). Isolation of taeniid eggs from faeces was performed by sieving/flotation technique, followed by DNA isolation, PCR and sequence analyses for species identification (gene target: small subunit of ribosomal RNA). Genetic diversity of *E*. *granulosus* *s*.*s*. was based on the sequence (1609 bp) of the *cox1* gene. A total of 67 out of 953 (7%) dog faecal samples were positive for at least one taeniid species. From the 670 free-roaming dog faecal samples, 40 (5.9%) were positive for taeniid DNA, 22 (3.2%) of them were identified as *E*. *granulosus* *s*.*s*. and four (0.5%) as *E*. *ortleppi* (G5). From the 283 faecal samples originating from yak-grazing areas, 27 (9.5%) were taeniid positive, including eight (2.8%) infected with *E*. *granulosus* *s*.*s*. and four (1.4%) with *E*. *ortleppi*. *E*. *granulosus* *s*.*s*. was identified in all isolates from human and the cyst from mithun. A haplotype network (*cox1* gene) from *E*. *granulosus* *s*.*s*, including isolates from 12 dogs, two human and one mithun, revealed eight different haplotypes. The most common *cox1* haplotype was the globally distributed Eg01, followed by Eg40 and Eg37 (previously described in China). Five new *cox1* haplotypes (EgBhu1–5) originated from human, dogs, and a mithun were identified. The study indicated the contamination of urban areas and pastures with *Echinococcus* eggs in seven districts in Bhutan. The molecular characterisation of *E*. *granulosus* *s*.*l*. revealed different *E*. *granulosus* *s*.*s*. haplotypes as well as *E*. *ortleppi*. The transmission of *T*. *multiceps* was documented only in the western part of the country. Considering the zoonotic feature of *E. granulosus s.s.* and *E. ortleppi* and the economic impact of coenurosis caused by *T. multiceps* (also known as gid) in Bhutan, the findings of this study represent a significant contribution towards an epidemiological baseline for the establishment of a national control programme.

## 1. Introduction

Bhutan has a large population of owned and stray dogs that cohabitate with humans. This situation is a concern for the general public. It is estimated that there are 71,245 owned and 48,379 stray dogs in the country, with a total human population of 727,145 inhabitants [1,2]. Although dogs are important companion animals, their role in the transmission of a diverse range of zoonotic infections to humans, including rabies and parasitic diseases, have to be considered [3,4]. Among various canine parasites in Bhutan, eggs of taeniids (*Taenia* and *Echinococcus*) have been documented in dogs by microscopic examination of faeces and molecular analysis [5].

The most recent taxonomic revision in the taeniid family recognises only four genera: *Hydatigera, Taenia, Versteria* and *Echinococcus* [6]. The species in the genus *Echinococcus* that causes cystic echinococcosis in intermediate and dead-end hosts including humans, (grouped as *E. granulosus sensu lato*), are *E. granulosus sensu stricto* (genotypes G1, G3 and microvariants), *E. equinus* (G4), *E. ortleppi* (G5), *E. canadensis* (G8, G10), and the proposed species *E. intermedius* (G6, G7) [7]. Cystic echinococcosis is a worldwide distributed neglected zoonoses [8] transmitted by *Echinococcus* eggs excreted in the faeces of mainly domestic dogs into the environment [9]. Cystic echinococcosis has a considerable disease burden and a significant medical impact caused by the space-occupying *Echinococcus* cysts, primarily in the liver and lungs [10]. In humans, *E. granulosus s.s*. is considered to be the most common species responsible for cystic echinococcosis, followed by G6 and G7 and G5 with a minor role in human infection. While G8, G10 [8,11,12] and recently, G4 [13,14] have been documented only in few cases.

A previous pilot study described *E. granulosus s.s.* in dogs, cattle and yaks, and *E. ortleppi* in cattle in Bhutan [5]. However, there was no information on the molecular characterisation of *E. granulosus s*.*l*. in human isolates. In this study, we identified *E. granulosus s.l* infecting humans and mithun, dogs from yak grazing areas and free-roaming and stray dogs in Bhutan. This study established a transmission pattern for *E. granulosus s.s*. Genetic diversity within *E. granulosus s.s.* was also investigated and presented in this study. Furthermore, we investigated the presence of *T. multiceps* in the yak grazing/pasture areas that reported coenurosis (gid disease) in yaks in Bhutan.

## 2. Results

A total of 67 out of 953 (7%) putative dog faecal samples were positive for at least one taeniid species (detailed information is shown in Table S1) through PCR. From the 670 faecal samples collected in urban and rural environments, 40 (5.9%) were positive for taeniid DNA; 22 (3.2%) of them were identified as *E. granulosus s.s.*, four (0.5%) as *E. ortleppi,* two (0.2%) as *T. multiceps* and 11 (1.6%) as various taeniid species. Table 1 shows the detailed description of the species found in all samples from free-roaming dogs. From the 283 dog faecal samples from yak rearing areas, 27 (9.5%) were taeniid positive, including eight (2.8%) identified to be infected with *E. granulosus* s.s., four (1.4%) with *E. ortleppi*, six (2.1%) with *T. multiceps* and eight (2.8%) as various taeniid species. All species identified in the dog faecal samples from yak grazing areas are shown in Table 2.

**Figure 1:**
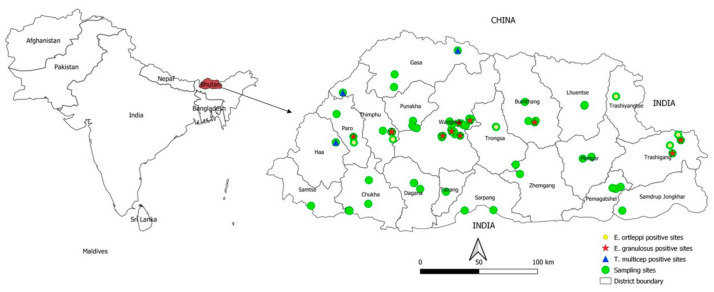
Geographic distribution of *Echinococcus granulosus s.s.*, *E*. *ortleppi and Taenia multiceps* isolated from dog faecal samples in Bhutan.

*T. multiceps* eggs were recovered in dog faecal samples from three yak rearing districts in western Bhutan (Gasa, Thimphu and Haa) (Figure 1). In contrast, *T. multiceps* was not detected in dog faecal samples from Trashigang (Merak-Sakteng) and Trashiyangtse (Bumdeling) yak rearing areas. Furthermore, various taeniid eggs were detected from 14 districts except for Chukha, Mongar, Pemagatshel, Punakha, Samdrup Jongkhar and Zhemgang (Table 1 and Table 2).

**Figure 2:**
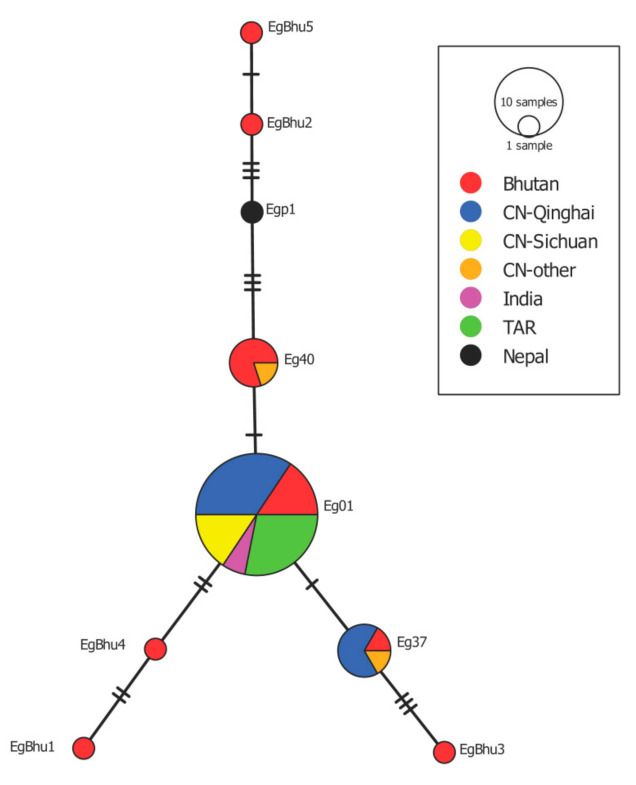
Haplotype network built with the sequence of the *cox1* gene of *E. granulosus s.s.* isolated from Bhutan named EgBhu1-EgBhu5 (accession numbers MW138944-MW138948) and similar sequences deposited in GenBank isolated from China (CH), India, TAR and Nepal. Eg01 (JQ250806) (5 samples from Bhutan, 11 from CH-Qinghai, two from India and nine from TAR), Eg37 (AB688614) (1 from Bhutan, four from CH-Qinghai and one from CH-other) and Eg40 (AB688617) (4 from Bhutan and one from CH-Qinghai).

Sequence analysis of the samples according to the results of multiplex PCR [15] and a section of the *cox1* gene [16] from 13 cysts from human CE patients and the mithun cattle (*Bos frontalis*) revealed the presence of *E. granulosus s.s*. Amplification of the 1609 bp of the *cox1* gene of *E. granulosus s.s*. for the haplotype diversity study was possible in 12 samples from dogs (5 from Wangdue, one from Bumthang and six from Trashigang), two from human and in one cyst from a mithun. Eight different *cox1* haplotypes of *E. granulosus s.s.* were identified in the 15 samples mentioned above. The most common haplotype was Eg01 in five samples from dogs, followed by the haplotype Eg40 found in four dog samples and the haplotype Eg37 found in one dog sample. Two sequences from dogs, two from human and one from a mithun, showed no 100% homology with similar entries in GenBank and were named EgBhu1-EgBhu5 (accession numbers MW138944-MW138948). EgBhu1 and EgBhu2 were found in human, EgBhu3 and EgBhu4 in dogs and EgBhu5 in the mithun. A haplotype network was built with the sequences found in this study and with sequences of the same length from neighbouring countries, including China, India, Tibetan Autonomous Region (TAR) and Nepal, deposited in GenBank (Figure 2). The *cox1* haplotype of *E. granulosus s.s.* Eg01 occupies the centre of the network together with isolates from India, China (Qinghai and Sichuan) and TAR. The newly described haplotypes from Bhutan (EgBhu1–5) differ between two and seven nucleotides with Eg01.

## 3. Discussion

This is the first nationwide detailed study conducted in Bhutan to investigate the prevalence of various taeniid species, particularly *E. granulosus s.l.* and *T. multiceps* in dogs. Our study revealed infections with *E. granulosus s.s.* in dogs and also in humans in the same districts of sampling, demonstrating the existence of active dog-human transmission. Furthermore, the presence of *E. granulosus s.s.* in infertile cysts in a mithun bull at Yusipang cattle breeding farm, Thimphu (Figure 1) suggests local transmission in the farm. In addition, in this study, *E. granulosus s.s.* was found in dog faeces from a sheep farm and in human cysts from Bhutan’s central part. Although there is no commercial-level slaughtering of sheep for human consumption due to the low number of sheep (*n* = 11,466) in Bhutan [17], it is essential to investigate in the near future the role that sheep play in the transmission of *E. granulosus*. Dogs accessing sheep carcass in waste disposals may likely have an association with the transmission. A previous pilot study in Bhutan demonstrated local transmission of *Echinococcus* spp. in dogs and the presence of *E. granulosus s.s.* and *E. ortleppi* in imported cattle from India and Nepal [5].

In Bhutan, there are no large-scale abattoirs and most of the meat (beef) consumed in the country is imported mostly from India with 3190 metric tonnes (MT) per year. National beef production for 2019 was just 410.7 MT. Sheep are reared primarily for wool and the slaughter of this species only occurs during the festive season and there is no official record for meat production for sheep [18,19]. It is reported that intestinal infections with *E. granulosus s.l.* are common among dogs in large parts of Asia, including northern India and Nepal [8] which is associated with improper livestock slaughter practices and free access of dogs to offal from slaughterhouses [20]. The situation in Bhutan is similar to the neighbouring countries, where meat hygiene practices are poorly followed in butcher shops. It is also common to see packs of community/stray dogs around butcher shops where they can have access to offal [21]. A control programme of the dog population was established in 2009 in collaboration with the Human International Society (HIS). From 2020, the national dog population management (NDPM) was established to improve dogs’ health and welfare and reduce the dog population. This initiative could reduce the transmission of *E*. *granulosus*. Additionally, the lack of awareness on the life cycle of *E. granulosus s.l.* and the inadequate implementation of legislation on meat inspection and the improper disposal of infected organs at meat shops contribute to the continuing transmission of the parasite [22].

Dispersion of taeniid eggs from the carnivore faeces through water or by adhering to objects (e.g., shoes and tires) and also by vectors such as birds and flies have been reported [23]. This will lead to further transmission of *Echinococcus* and increase the risk of human infections by hand-mouth contact after exposure to an *Echinococcus* contaminated environment or by consuming contaminated food [18,24]. At present, no epidemiological data allows the quantification of such infection risks [12,18]. Considering that *E. granulosus s.s.* is zoonotic, the findings of this study may be used as a basis to strengthen the prevention and control of infection in dogs, thereby preventing CE in humans. The study’s limitations are mainly based on the difficulties to document sources of infections for the free-roaming dogs, quantifying the importance of scavenging of dead animals on pastures and forests, and free access to infected meat waste on markets, meat shops and from home slaughtering. Therefore, further, more focussed studies are needed.

The analysis of the genetic diversity of *E. granulosus s.s.* was possible only in 15 samples, including just two human samples. Unfortunately, the preservation of human cysts in formalin 10% precluded us from amplifying the 1609 bp of the *cox1* gene in most of these isolates. The haplotype network shows the *cox1* variant called Eg01 in the central position as it has been previously described in Iran, China, Jordan, Peru, Australia, Chile and Bolivia [25,26,27,28]. The central location of this haplotype suggested that this is an ancient variant of the parasite, which was spread worldwide at the time of domestication of livestock as described by Yanagida et al. [25]. This haplotype is also present in the neighbouring TAR in China and India, perhaps suggesting how this variant spread in the past. Another haplotypes, Eg40 and Eg37, were also described in isolates from Bhutan, Nepal and China. The finding of five not previously described *cox1* haplotypes of *E. granulosus s.s.* in Bhutan is interesting for phylogeography studies. Perhaps it is indicative of random mutations accumulating over time and these variants established locally. It is important to keep in mind that the variability within *E*. *granulosus s.s.* is even higher when analysing the almost complete mitogenome of isolates [29]. However, it remains unclear if the nucleotide variability in mitochondrial genes within *E*. *granulosus s.s.* is relevant for the parasite’s biological features, which can produce differences in the development of the disease in humans and animals.

Another objective of this study was to investigate the presence of *T. multiceps* in the yak grazing/pasture areas that have reported coenurosis. Currently, coenurosis is being reported in yaks from the west and central region (Haa, Paro, Thimphu, Gasa and Bumthang districts) but it has not been reported in the yaks from the eastern part (Merak-Sakteng, Trashiyangtse and Lhuentse) [30]. Besides, Gangtey, Phobji and Sephu yak rearing areas in Wangdue Phodrang that previously reported coenurosis disease in yaks had controlled the disease since 2008 through the reduction of yak-dogs and regular deworming of both stray and yak-dogs, leading to no cases being reported in the mentioned areas. Our results confirm the findings of national surveillance data published in 2016 [30] and demonstrates that *T. multiceps* could not be recovered from dog faecal samples collected from yak rearing areas from the eastern regions with a historical absence of coenurosis. One of the main caveats of this study was the inability to collect faecal samples directly from yak-herding dogs due to logistical constraints and the remoteness of the yak herds. Samples from yak rearing areas were collected directly from the pasture. Therefore, we could not exclude that some of the samples originated from wild carnivores (e.g., foxes, wolves, or others).

## 4. Materials and Methods

### 4.1. Dog Faeces

From May 2016 to April 2018, 953 dog faecal samples were collected from the environment covering all the 20 districts of Bhutan. Dog faeces were identified based on their physical features, but no molecular discrimination was performed to unequivocally identify the host. From the 953 total samples, 670 faeces originated mainly from the centres of village settlements and towns where there is a high population of owned and free-roaming/stray dogs; and 283 faecal samples were collected from yak herder’s settlement and grazing pastures located up to 4500 m above sea level (masl). Faecal samples were collected into a vial and preserved using 70% ethanol. The samples were shipped to the National Centre for Animal Health (NCAH), Thimphu and stored at −20 °C until further analysis.

### 4.2. Taeniid Egg Isolation and Microscopic Identification

Approximately three grams of faeces from each sample were processed using flotation with saturated sugar solution (ratio 1:1) and sequential sieving with nylon mesh of decreasing sizes of 105 µm, 40 µm and 21 µm (Lanz-Anliker AG, Allmendstrasse 12, 4938 Rohrbach, Switzerland). This method allowed taeniid eggs to be concentrated in the last sieve, as described by Mathis et al. [31]. PET bottles, a cost-efficient and readily available material, were used as containers and funnels in a system adapted by Peter Deplazes (Figure S1). The funnel was created by cutting the PET bottles into two uneven parts, the part with the cap serving as a funnel. A round hole was cut into the centre of the caps to allow the nylon meshes’ insertion to be used as sieves. The bottom part of each bottle was used as a container to hold the filtrate after passing all filters. Sediments from the 21 µm sieves were then collected and microscopically examined. Those positive for taeniid eggs (Figure S2a) were stored at −80 °C until further referral to the Institute of Parasitology, University of Zurich, Switzerland for molecular analysis. Sieving materials from positive samples were discarded to avoid cross-contamination. To prevent egg and DNA contamination between the samples, all sieving materials used in samples that were negative for cestode eggs were incubated in sodium hypochlorite (1% active chlorine concentration) for at least 30 min and subsequently washed before reuse.

### 4.3. Cystic Echinococcosis Cysts

A total of 13 human cystic echinococcosis cysts were collected between 2015 and 2017 from 13 patients undergoing surgery at the Jigme Dorji Wangchuck National Referral Hospital (JDWNRH), Thimphu, Bhutan. The patients originated from Bumthang (3 cases), Paro (1), Wangdue Phodrang (1), Phuentsholing (1) and Samtse (2) and unknown locations (5) (Figure 1). The intact cyst samples were preserved in 10% formalin at JDWNRH before being sent to the laboratory at NCAH. The samples were washed four times using distilled water. Cyst adventitia was then opened, and the parasite material collected from hydatid cysts was placed on a 1 mm size mesh sieve, scraped and washed several times with distilled water. About 1.5 mL of sediment containing protoscoleces or cellular material (Figure S2b) were preserved in 70% ethanol and stored at −80 °C at NCAH for genetic characterisation. Cysts from the lungs and spleen (Figure S2c,d) were collected during post mortem examination of a mithun breeding bull from the National Dairy Research Centre, Yusipang, Thimphu. The necropsy took place at NCAH, Serbithang, Thimphu, in April 2017. Isolation of parasite tissue was performed as described above for human cysts but the sample was directly preserved in 70% ethanol.

### 4.4. Molecular Analysis

#### 4.4.1. Dog Faecal Samples

The positive samples for taeniid eggs were subjected to DNA extraction as described previously [32] and multiplex polymerase chain reaction (PCR) using mitochondrial DNA targets (small subunit of ribosomal RNA) for identification of *Echinococcus* species and other cestodes, including *Taenia* spp. using a multiplex PCR kit (Qiagen, Hilden, Germany) [15]. The amplified PCR products were purified using the MinElute PCR purification kit (Qiagen, Hilden, Germany) according to the manufacturer’s instructions and then sequenced as previously described [15] at Microsynth, Switzerland. Sequences were compared with the GenBank nucleotide database, using BLAST search (http://www.blast.ncbi.nih.gov, accessed on 20 November 2020).

#### 4.4.2. Human and Bovine Cyst Samples

Total DNA was extracted from each cyst sample of human (*n* = 13) and one mithun origin using the tissue protocol described in the Qiagen Mini Kit (Qiagen, Hilden, Germany). All samples were subjected to PCR and sequenced, according to Trachsel et al. [15]. Confirmation of the parasite species responsible for the infection was accomplished by PCR and sequencing of a section of the *cox1* gene (366 bp), according to Bowles et al. [16]. Sequencing results were compared with entries in GenBank as described above.

#### 4.4.3. Haplotype Diversity within *E. granulosus s.s.*

Total DNA from samples identified as *E*. *granulosus s*.*s*. from dogs, humans and mithun was used as a template for the amplification and sequencing of 1609 bp of the *cox1* gene as previously described [25]. Only sequences with single peaks in electropherograms were included in this study. Sequences were compared with all similar sequences of the same length deposited in GenBank. A haplotype network was built using PopArt [33] with sequences acquired in this study and similar sequences from countries sharing borders with Bhutan.

## 5. Conclusions

Our study documents the presence of *E. granulosus s.s.* in environmental dog faeces and in human cystic echinococcosis cysts in the same districts indicating ongoing disease transmission. Furthermore, the detection of *E. granulosus s.s*., *E. ortleppi*, *T. multiceps*, and other *Taenia* spp. from community and yak dogs faeces indicates environment contamination of various taeniid eggs in urban and rural habitats. The findings from this study suggest salient interventions which could be implemented in the future, particularly: (a) implementation of regular deworming of community, private and yak-herding dogs, (b) restrictions in movement of yaks and dogs from coenurosis endemic region to coenurosis free regions, (c) a need to improve food hygiene, slaughter process inspection, and adequate disposal of the offal (d) enhance the engagement of public health authority to reduce the incidence of human cystic echinococcosis.

## Figures and Tables

**Table 1:** Origin of 670 environmental faecal samples of free-roaming dogs in Bhutan (district level), including the molecular species identification of taeniid eggs.

District	Samples with Taeniid Eggs/Total Number Investigated	Taeniid Species Identified (Number of Samples)
Bumthang	3/50	*E. granulosus s.s*. (3)
Chukha	0/61	*-*
Dagana	1/30	*Hydatigera taeniaeformis* (*Syn. Taenia taeniaeformis*) (1)
Gasa	0/36	*-*
Haa	2/16	*T. multiceps* (2)
Lhuentse	3/26	*H. taeniaeformis* (3)
Mongar	0/10	*-*
Paro	5/28	*E. granulosus s*.*s*. (3) *E. ortleppi* (2)
Pemagatshel	0/37	*-*
Punakha	0/55	*-*
Samdrup Jongkhar	0/26	*-*
Samtse	1/35	*T. hydatigena* (1)
Sarpang	2/26	*T. hydatigena* (2)
Thimphu	3/34	*E. granulosus s.s.* (2) *E. ortleppi* (1)
Trongsa	1/24	*E. ortleppi* (1)
Tsirang	1/23	*T. hydatigena* (1)
Wangdue Phodrang	18/143	*E. granulosus s.s.* (14) *H. taeniaeformis* (2) *Taenia* spp. (1) No specific match (1)
Zhemgang	0/16	-
Total	40/670	

**Table 2:** Origin of 283 environmental faecal samples of free-roaming dogs in Bhutan (district level) from yak herder’s settlements and yak grazing pastures, including the molecular species identification of taeniid eggs.

District	Samples with Taeniid Eggs/Total Number Investigated	Taeniid Species Identified (Number of Samples)
Bumthang	2/20	*H. taeniaeformis* (2)
Gasa	4/15	*T. multiceps* (4)
Thimphu	2/57	*T. multiceps* (2)
Trashigang	17/154	*E. granulosus s.s.* (8) *E. ortleppi* (3) *T. hydatigena* (4) *T. ovis* (1) No specific match (1)
Trashiyantse	2/37	*E. ortleppi* (1) *H. taeniaeformis* (1)
Total	27/283	

## Data Availability

The supplementary data presented in this study is available in the present article.

## Supplementary Materials

The following are available online at https://www.mdpi.com/2076-0817/10/3/330/s1, Figure S1: Modified sieving method using plastic one-way water bottles for the sieving step of the supernatant after centrifugation of the faecal suspension, Figure S2 (a) Taeniid eggs isolated by Floatation and Sedimentation method from dog faeces, which were later identified as *Taenia multiceps*, causing coenurosis in yaks; (b) Protoscolex of *Echinococcus granulosus* from a human cyst; (c) Cysts in mithun lungs, and (d) Cysts of *E. granulosus* s.s. in a mithun spleen, Table S1 Detailed sampling sites and genetic identification of eggs of various taeniid species in Bhutan.
