# Supplementary material for: Occurrence of Echinococcus granulosus sensu lato and Other Taeniids in Bhutan
Source: Pathogens. 2021 Mar 11;10(3):330. doi: 10.3390/pathogens10030330 (PMC8001613; doi:10.3390/pathogens10030330)
Supplement: Supplementary file 1 [file pathogens-10-00330-s001.pdf]

Table S1. Detailed information of sampling sites, number of samples and genetic identification of eggs of various taeniid species in Bhutan.

District	Sampling sites	Dog type	Total sample	No. positives	Species
Bumthang	Bumthang town	Stray dog	27	0	-
	National Sheep Breeding Farm	Stray dog	12	3	*E. granulosus s.s.*
	DechenpelriDratshang	Stray dog	11	0	-
	Choekor (Yak rearing area)	Stray dog/Yak dog*	20	2	*Hydatigera taeniaeformis* (*Syn. Taenia taeniaeformis*)
Chukha	Phuentsholing lower market	Stray dog	7	0	-
	Phuentsholing bus station	Stray dog	8	0	-
	Phuentsholing football stadium	Stray dog	8	0	-
	Gedu town	Stray dog	13	0	-
	Tshimakothi town	Stray dog	25	0	-
Dagana	Dagana town	Stray dog	20	0	-
	Dagana hospital area	Stray dog	5	0	-
	DHPC colony	Stray dog	5	1	*H. taeniaeformis*
Gasa	Gasa town	Stray dog	30	0	-
	Damji	Stray dog	6	0	-
	Lunana	Stray dog/Yak dog*	15	4	*T. multiceps*
Haa	Haa town	Stray dog	16	2	*T. multiceps*
Lhuentse	Lhuentse town	Stray dog	12	2	*H. taeniaeformis*
	Tunkulung hospital area	Stray dog	8	1	*H. taeniaeformis*
Mongar	Lingmethang town	Stray dog	5	0	-
	Mongar Livestock campus	Stray dog	5	0	-
Paro	Paro Market area	Stray dog	20	3<br>2	*E. granulosus s.s.*<br>*E. ortleppi*
	Lango	Stray dog	8	0	-
Pemagatshel	Zobel area	Stray dog	15	0	-
	Pemagatshel hospital area	Stray dog	6	0	-
	Pemagastshel School area	Stray dog	11	0	-
	Nangkhor school	Stray dog	5	0	-
Punakha	Khuruthang	Stray dog	37	0	-
	Lobesa	Stray dog	6	0	-
	CNR campus	Stray dog	12	0	-
Samdrup Jongkhar	Dewathang town	Stray dog	26	0	-
Samtse	Samtse town	Stray dog	35	1	*T. hydatigena*
Sarpang	Sarpang town	Stray dog	14	0	
	Gelephu town	Stray dog	12	2	*T. hydatigena*

Thimphu	Thimphu city	Stray dog	10	0	-
	Yusipang NDRC	Stray dog	3	1	*E. granulosus s.s.*
	Yusipang Residential	Stray dog	5	1	*E. granulosus s.s.*
	Yusipang office area	Stray dog	5	1	*E. ortleppi*
	Yusipang cattle shed area	Stray dog	6	0	-
	Yusipang piggery office area	Stray dog	5	0	-
	Soe	Stray dog/Yak dog*	44	2	*T. multiceps*
	Gunitsawa	Stray dog/Yak dog*	13	0	-
Trashigang	Merak village	Stray dog/Yak dog*	54	5	*E. granulosus s.s.*
				2	*E. ortleppi*
				3	*T. hydatigena*
	Sakteng village	Stray dog/Yak dog*	100	3	*E. granulosus s.s.*
				1	*E. ortleppi*
				1	*T. hydatigena*
				1	*T. ovis*
				1	no specific match
Trashiyangtse	Tarphel, Bumdeling	Stray dog/Yak dog*	37	1	*E. ortleppi*
				1	*H. taeniaeformis*
Trongsa	Sunday Market area	Stray dog	21	1	*E. ortleppi*
	Sherubling	Stray dog	3	0	-
Tsirang	Damphu town	Stray dog	23	1	*T. hydatigena*
Wangdue Phodang	Bajo town	Stray dog	31	0	-
	Nobdling	Stray dog	11	1	no specific match
	Gangtey temple	Stray dog	14	1	*H. taeniaeformis*
	Gantey	Stray dog	10	0	-
	Phobjekha School area	Stray dog	13	7	*E. granulosus s.s.*
	Lawala Pass	Stray dog	2	0	-
	Darjizab village	Stray dog	2	0	-
	Khewang	Stray dog	5	0	-
	Tabading	Stray dog	5	1	*E. granulosus s.s.*
	Chupja	Stray dog	5	0	-
	Gogojikha	Stray dog	5	1	*H. taeniaeformis*
	Lhakhangtsawa	Stray dog	5	2	*E. granulosus s.s.*
	Busa	Stray dog	5	3	*E. granulosus s.s.*
	Zeeri	Stray dog	5	0	-
	Lupzor	Stray dog	5	0	-
	Rukubji	Stray dog	5	0	-
	Bumilo	Stray dog	5	1	*Taenia* spp.

	Longtey	Stray dog	5	1	*E. granulosus s.s.*
	Longmey	Stray dog	5	0	-
Zhemgang	Zhemgang town	Stray dog	5	0	-
	Nangkhor	Stray dog	5	0	-
	Tingtibi	Stray dog	6	0	-
	**Total**		**953**	**67**	

*** The faeces samples collected can be from stray dogs/yak dogs but from yak grazing/pasture areas which are approximately 4,500 masl.**

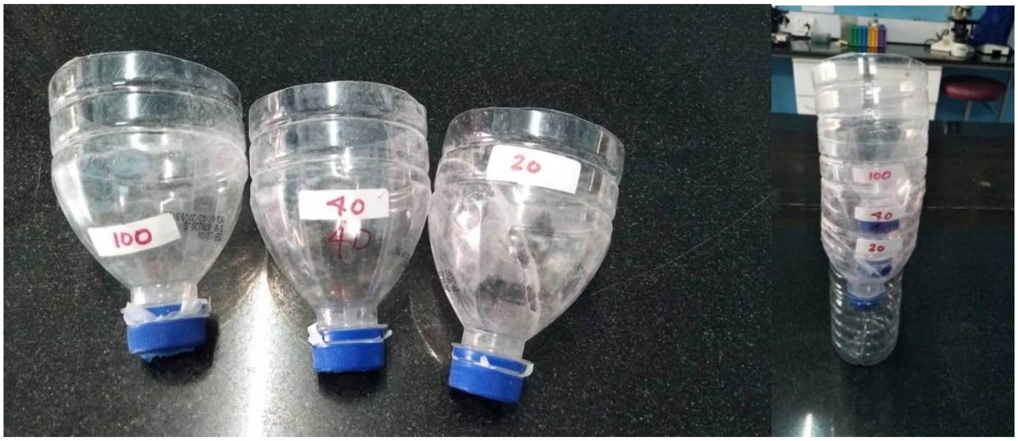

Fig. S1 Modified sieving one-way method using PET bottles for the sieving step of the supernatant after centrifugation of the faecal suspension. Numbers 100, 40 and 21 indicate the pore size of the different nylon meshes used.

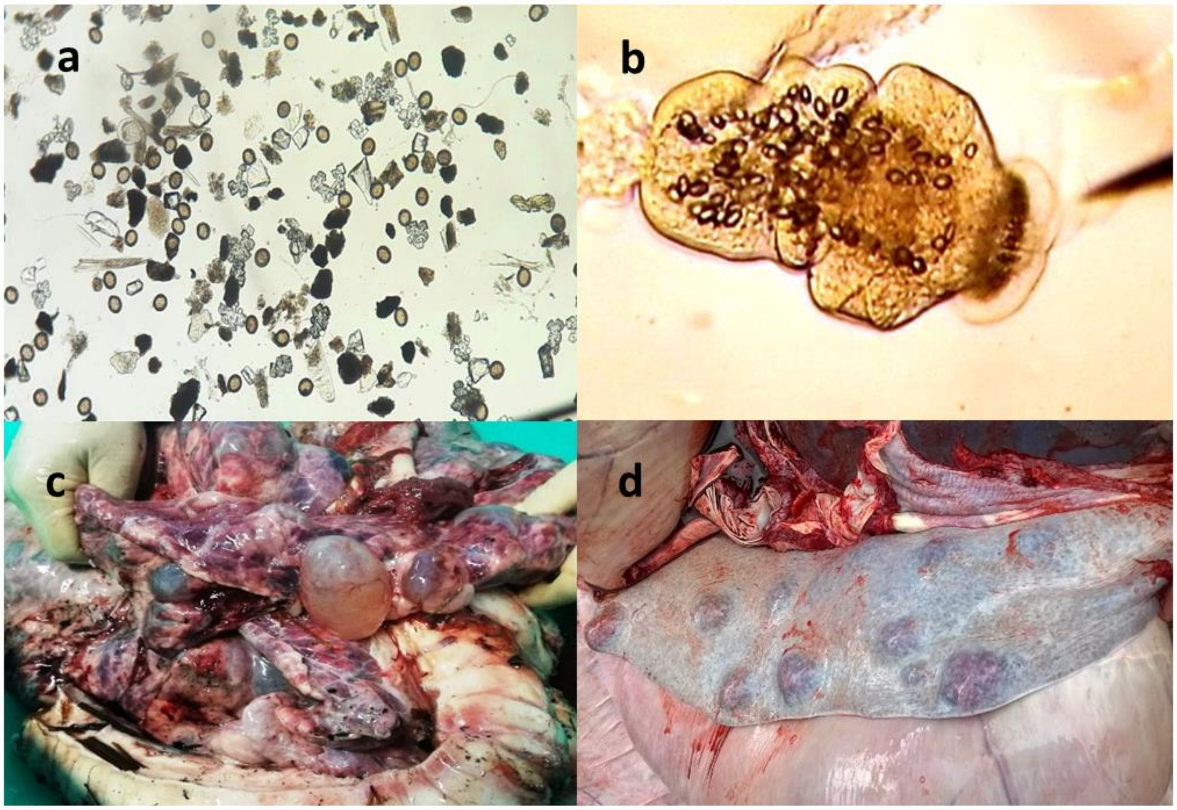

Figure S2: a) Taeniid eggs isolated by floatation and sedimentation method from dog faeces, which were later identified as *Taenia multiceps*; b) protoscolex of *Echinococcus granulosus* from a human cyst; c) cysts in mithun lungs, and d) cysts of *E. granulosus* in a mithun spleen.
